# Regulatory problems and associated factors among infants in Arba Minch health and demographic surveillance system sites, southern Ethiopia

**DOI:** 10.1371/journal.pone.0305722

**Published:** 2024-06-18

**Authors:** Agegnehu Bante, Gistane Ayele, Birhanu Alamirew, Muluken Ahmed

**Affiliations:** 1 School of Nursing, College of Medicine and Health Sciences, Arba Minch University, Arba Minch, Ethiopia; 2 School of Public Health, College of Medicine and Health Sciences, Arba Minch University, Arba Minch, Ethiopia; 3 School of Medicine, College of Medicine and Health Sciences, Arba Minch University, Arba Minch, Ethiopia; University of Peradeniya, SRI LANKA

## Abstract

**Introduction:**

Infant regulatory problems are a common source of concern for parents, and they increase the risk of impaired infant-caregiver bonding. Despite their impact, they are often overlooked in Ethiopia. Hence, this study aimed to determine the prevalence and associated factors of infant regulatory problems in Arba Minch Health and Demographic Surveillance System sites in southern Ethiopia.

**Methods:**

A community-based cross-sectional study was conducted among 451 mother-infant pairs from February 15 to March 15, 2022. Regulatory problems were assessed using diagnostic interviews for regulatory problems. The data was collected using an open data kit Android application and analyzed with Stata version 17.0. Bivariable and multivariable logistic regression analyses were computed to identify factors associated with each infant regulatory problem. Statistical significance was declared at a p-value < 0.05.

**Results:**

In this study, four hundred forty-nine mother-infant pairs were involved, with a response rate of 99.5%. The prevalence of excessive crying, feeding problems, and sleeping difficulties was 14.03% [95% CI: 10.95, 17.59], 20.04% [95% CI: 16.44, 24.05], and 13.59% [95% CI: 10.55, 17.11], respectively. Attending primary education (AOR: 2.54, 95% CI: 1.22, 5.32), high perceived social support (AOR: 0.32, 95% CI: 0.12, 0.89), feeding problems (AOR: 5.0, 95% CI: 2.65, 9.45), and depression, anxiety, and stress (DAS) symptoms (AOR: 2.67, 95% CI: 1.19, 5.98) were associated with excessive crying. In addition, a family size of above five (AOR: 1.82, 95% CI: 1.03, 3.22), excessive crying (AOR: 3.76, 95% CI: 1.85, 7.65), sleeping problems (AOR: 2.29, 95% CI: 1.13, 4.65), comorbid DAS symptoms (AOR: 3.42, 95% CI: 1.64, 7.11), alcohol abuse (AOR: 1.89, 95% CI: 1.04, 3.42), and late initiation of complementary feeding (AOR: 2.67, 95% CI: 1.22, 5.88) were associated with feeding problems. Furthermore, attending primary education (AOR: 2.35, 95% CI: 1.16, 4.77), feeding problems (AOR: 3.47, 95% CI: 1.86, 6.48), and comorbid DAS symptoms (AOR: 3.23, 95% CI: 1.53, 6.84) were associated with sleeping problems.

**Conclusions:**

Approximately one-third of infants encountered at least one regulatory problem. Level of education, perceived social support, feeding problems, and DAS symptoms were associated with excessive crying. Large family sizes, excessive crying, sleeping problems, comorbid DAS symptoms, alcohol abuse and, late initiation of complementary feeding increase the likelihood of feeding problems. Moreover, attending primary education, feeding problems, and comorbid DAS symptoms increase the odds of sleeping problems. Continuous guidance and support on infant soothing techniques, cognitive and behavioral therapy, and counselling on appropriate coping strategies for postpartum women are imperative to reduce the burden of infant regulatory problems.

## Introduction

Infant regulatory problems (RPs) are defined as excessive crying, sleeping, and feeding problems that occur during the first year of life [1]. Infant RPs are an additional hardship for parents and may happen as isolated or multiple/comorbid problems [2]. RPs, especially their comorbidity, can increase the risk of developmental and mental health problems throughout childhood and adulthood [3]. Even though the exact mechanism is unknown, an immature nervous system, altered circadian rhythm, and intestinal microorganisms significantly contribute to infant RPs [4].

Infant RPs are a significant public health concern worldwide; 25% and 4–10% of infants experience isolated and comorbid RPs, respectively [2]. Although their prevalence in developing countries is unclear, it ranges from 5.3 to 38.5% in industrialized countries [3, 5–7]. Excessive crying alone is a reason for ~20% of health facility visits [8]. Moreover, Cook and colleagues also discovered that 25.4%, 13.2%, and 3.4% of 1759 infants had sleeping problems, complex regulatory difficulties, and severe regulatory difficulties, respectively [9].

Infant RPs may indicate a severe underlying medical condition that results in a long-term sequel [4]. Bilgin and coworkers found that early RPs during infancy increase the risk of attention deficit hyperactivity disorder (ADHD) in childhood and adulthood [10]. Cook and co-authors also reported that infants with moderate and severe RPs have three- and five-times higher odds of language difficulties at five and eleven years, respectively [11]. In addition, RPs increase the likelihood of childhood behavioral disorders, conduct, hyperactivity, and mood disorders in later life [12, 13]. RPs also increase the risk of clinically significant mental health disorders during childhood that worsen over time [9]. The impacts of RPs are not limited to infants; they lead to various parental problems [13, 14]. They impair maternal-infant bonding, exacerbate behavioral problems, increase parental stress, impede breastfeeding, and potentially incite relationship breakdowns [14–16]. Furthermore, infant RPs increase the odds of maternal DAS, doubt, anger, poor sleeping quality, and inadequate quality of life [8, 17, 18].

Previous studies consistently reported that parental bedtime behaviors, low maternal schooling, single family, young maternal age, primiparity, and intense antenatal bonding increase the odds of infant RPs [3, 6, 19–21]. Furthermore, parent cognition problems, poor parental cry tolerance (PCT) and parental distress, leaving the infants to cry, negative maternal mood and body contact, low maternal self-esteem, psychosocial stress, maternal anxiety and depression, breastfeeding, poor soothing techniques, non-reassuring fetal status, fecal microbiota, prematurity, and fetal abnormalities all increases the likelihood of infant RPs [5, 7, 20, 22–30].

Scholars suggested that continuous guidance and support, soothing techniques, PCT, and lower parental distress attribution are critical to minimize infant RPs [23, 27, 31]. However, many parents do not know about these problems; Tsai and coauthors reported that 15% of parents did not know their infant’s sleep was problematic [32]. There is also a knowledge gap regarding the interventions given for infants with RPs, and more evidence is mandatory to deliver evidence-based interventions [33].

Scholars have indicated that RPs in the first few months are considered an early adjustment to childhood development [34]. However, previous studies included infants younger than six months and assessed RPs using a tool with low psychometric properties. One of the few tools available to assess infant RPs is the diagnostic interview for regulatory problems (Baby-DIPS); it has strong inter-rater reliability and three subscales to assess excessive crying, feeding, and sleeping problems [35].

Although RPs are the most frequent reason for a healthcare facility visit during early childhood and a source of parental distress, studies have been limited to developed countries [2, 5, 36–38]. As far as our search is concerned, this is the first study that addresses RPs among infants in Ethiopia. Understanding the prevalence and associated factors of infant RPs is critical for developing appropriate intervention strategies to reduce the burden and lifetime sequelae of these problems. As a result, this study aimed to determine the prevalence and associated factors of RPs among infants in Arba Minch health and demographic surveillance sites in southern Ethiopia.

## Methods

### Study design, setting, and period

A community-based cross-sectional study was conducted among infants in Arba Minch Health and Demographic Surveillance System (AM-HDSS) sites from February 15 to March 15, 2022. The surveillance site is in the Arba Minch Zuria district, with the administrative center of Arba Minch Town (the capital of Gamo Zone) located 505 km (about 313.79 mi) south of Addis Ababa (the capital city of Ethiopia). The surveillance activities were implemented in 9 kebeles (Ethiopia’s smallest administrative units) based on population proportion to size, attitude, service accessibility, and urban and rural composition. Eight of the kebeles are rural, while the other is semi-urban. According to the HDSS 2023 report, the surveillance site had a total population of 88,078 (43,883 females) and 17,588 households.

### Study population

All mothers/guardians with six-to-twelve-month infants living in AM-HDSS were considered as the study population and included in this study. Mothers/guardians with severe mental illness were excluded.

### Sample size determination and sampling technique

The sample size was determined by Epi-Info 7 StatCalc using a double population formula by considering the following assumptions: 95% level of confidence, 80% power, the proportion of excessive crying among singles (19.35%, P1) and among married mothers (8.84%, P2) [20], and being unexposed to an exposed ratio of 2. After adding a 10% non-response rate, the final sample size was 451 mother-infant pairs. Infants were identified in the kebeles with the help of the surveillance site registry and health extension workers. Then, the sample size was allocated proportionally based on the number of infants in each kebeles. Finally, using the AM-DHSS household registry as a sampling frame, simple random sampling technique was used to select the individual households with infants.

### Data collection tool

An interviewer-administered questionnaire (S1 File) was organized after reviewing previous literature [2, 5, 10, 15, 29]. The tool comprises six parts: Part one includes: socio-demographic and socioeconomic status such as maternal age, educational level, marital status, and occupation. The family’s economic status was collected by asking for ownership of selected assets common in the local area. Part two includes obstetric characteristics such as gravidity, parity, history of stillbirth, history of abortion, ANC follow-up, status of pregnancy, skin-to-skin contact, soothing techniques, breastfeeding, and complementary feeding practice. Part three includes infant-related variables such as sex, gestational age during delivery, sepsis, perinatal problems, and birth weight. Part four comprises infant regulatory problems, which include excessive crying, sleeping problems, and feeding difficulty. Infant RPs were assessed using diagnostic interviews for regulatory problems (Baby-DIPS), which has 13 items. Popp and colleagues revealed that the Baby-DIPS has good to excellent inter‑rater reliability in assessing infant regulatory problems (k: 0.77–0.98) [35]. Excessive crying was assessed using three items: crying for ≥ 3 hours/day, on ≥3 days/week, for ≥ 3 weeks consecutively. Feeding problems were assessed using five items: any feeding problem(s) from a list of 16 potential problems, failure-to-thrive, and mothers worrying about infant growth, these problems occurred for ≥ 4 weeks and were not better attributable to a concurrent medical condition. Likewise, sleeping problems were assessed using 5 items and defined as difficulties in initiating and maintaining sleep for ≥ 3 nights/week for ≥ 3 months while the mother was impaired by her infant’s sleeping difficulties [38].

Part five was about perceived social support (PSS) and domestic violence (DV). PSS was assessed using the multidimensional scale of perceived social support (MSPSS). MSPSS is a seven-point: 1 (very strongly disagree) to 7 (very strongly agree) Likert scale with 12 items. MSPSS is a reliable (Cronbach’s α-value of 0.933) measure of PSS [39]. DV was measured using five items on the Women’s Abuse Screening Test (WAST) scale [40]. Part six includes maternal DAS and alcohol abuse. Maternal DAS was assessed using the Depression, Anxiety, and Stress Scale (DASS-21), a set of three self-report scales designed to measure the emotional states of depression, anxiety, and stress. Each of the three DASS-21 scales contains 7 items, divided into three subscales to assess depression, anxiety, and stress separately. Scores for the DASS-21 sub-scales of DAS were derived by summing up the sub-scales and multiplying them by two to ensure the interpretation of the 42 items of the DASS [41]. Although not validated in the Ethiopian context, DASS is validated to measure common mental disorders in women with a Cronbach’s alpha of 0.88 [42]. Furthermore, alcohol abuse was measured using the fast alcohol screening test (FAST) scale, which has four questions [43]. The tool was organized in XLSForm in Excel and converted to XForms to collect the data electronically using Open Data Kit (ODK) tools.

### Data collectors and data collection procedure

Nine experienced data collectors and three field supervisors working for AM-HDSS were recruited for the data collection. Both theoretical and practical training was given to the data collection team for two consecutive days. ODK Collect version 1.17.2 android application was used to collect the data electronically. The tool was pre-tested on 23 mother-infant pairs from Arba Minch town two weeks before the data collection. Then the data was collected on the participants’ own after getting their informed consent. Supervisors and investigators contacted the data collectors regularly to check the data collection procedure. Finally, the data collectors sent the completed forms to the ODK aggregate server of AM-HDSS.

### Operational definitions

#### Regulatory problems

Those infants crying for ≥ 3 hours/day, ≥ 3 days/week, and for ≥ 3 weeks consecutively were categorized as excessive crying. Infants with any feeding problem(s) from a list of 16 potential problems (S1 File), failure-to-thrive, and mothers worrying about infant growth; these problems occurred for ≥ 4 weeks and were not better attributable to a concurrent medical condition were categorized as feeding problems. Likewise, sleeping problems were defined as difficulties initiating and maintaining sleep for ≥ 3 nights/week for ≥ 3 months while the mother was impaired by her infant’s sleeping challenges [38].

#### Perceived social support

It was assessed using MSPSS and its score ranges from 12–84; and was categorized into three, a score of 12–35 considered low, 36–60 as medium, and 61–84 as high PSS [39].

#### Domestic violence

Measured using the WAST scale, which ranges from 0–16, where a score > 1 indicates the presence of violence [40].

#### Depression, anxiety, stress

Women were categorized based on the recommended scoring and cut-off values of DASS-21. Normal (0–9 for depression, 0–7 for anxiety and 0–14 for stress), mild (10–13 for depression, 8–9 for anxiety and 15–18 for stress), moderate (14–20 for depression, 10–14 for anxiety and 19–25 for stress), severe (21–27 for depression, 15–19 for anxiety and 26–33 for stress), and extremely severe (≥ 28 for depression, ≥ 20 for anxiety and ≥ 34 for stress) [41].

#### Alcohol abuse

It was measured using the FAST scale with a score ranging from 0 to 16, where a score ≥ 3 indicates hazardous drinking [43].

### Data analysis

The data set was downloaded from the AM-HDSS aggregate server as an Excel file and imported to Stata version 17.0 for analysis. Descriptive statistical analyses were used to describe participants’ characteristics. Infant RPs were categorized in to five based on the presence or absence of the RPs: free from regulatory problems, isolated crying, isolated feeding, and isolated sleeping, multiple RPs (combined crying and feeding, crying, and sleeping, feeding, and sleeping), and comorbid (simultaneous occurrence of crying, sleeping, and feeding problems) [5, 7]. The family wealth index was computed using principal component analysis (PCA) and grouped into three quintiles. Bivariable analysis with crude odds ratio was used to see the association between independent variables and excessive crying, feeding problems and sleeping difficulties. To control confounding, variables with a p-value of < 0.25 in the bivariable analysis were taken to the multivariable analysis for each regulatory problem. The Hosmer-Lemeshow test and variance inflation factor (VIF) were run for each model to check the model goodness of fit and multicollinearity, respectively. An odds ratio with 95% CI was used to identify factors associated with each infant RP in the multivariable analysis. A p-value of < 0.05 was set to declare statistical significance.

### Ethical considerations

Arba Minch University, College of Medicine and Health Sciences, Institutional Review Board (IRB) provided ethical approval for this study with a protocol number of IRB/1178/2021. Permission was also obtained from each kebeles administration. Moreover, this study followed the Declaration of Helsinki; written informed consent was secured from the mothers/guardians. Code numbers were used throughout the study to maintain the confidentiality of information gathered from each participant. Infants with RPs were addressed through the household number recorded during data collection, and home-based counselling services about the soothing techniques were given to the parents. Furthermore, they were linked to the health extension workers and local health facilities for further investigation and treatment.

## Results

### Maternal sociodemographic characteristics

In this study, 449 mother-infant pairs were involved, with a response rate of 99.5%. The mean age of the mothers was 29.08 years (SD: 5.48). Two-fifths (38.08%) were between 25–29 years old, and most (95.55%) were married. Regarding educational status, more than half (51.45%) could not read and write. The majority (80.18%) were housewives in their occupation, rural dwellers (78.84%), and a family size of five and below (50.33%). Over one-third (35.41%) had medium socioeconomic status (Table 1).

**Table 1:** Socio-demographic characteristics of participants in Arba Minch health and demographic surveillance system sites, southern Ethiopia, 2022 (n = 449).

Variables	Category	Frequency	Percentage
Age (in completed years)	< 25	84	18.71
	25–29	171	38.08
	30–34	117	26.06
	> 34	77	17.15
Marital status	Single	7	1.56
	Married	429	95.55
	Divorced	5	1.11
	Living apart	8	1.78
Educational status	Unable to read & write	231	51.45
	Able to read and write	51	11.36
	Primary education	99	22.05
	Secondary education	49	10.91
	College and above	19	4.23
Maternal occupation	Housewives	360	80.18
	Farmer	54	12.03
	Merchant	11	2.45
	Government employee	8	1.78
	^a^Other	16	3.56
Residence	Semi-urban	95	21.16
	Rural	354	78.84
Family size	≤ 5	226	50.33
	> 5	223	49.67
Wealth index	Low	144	32.07
	Medium	159	35.41
	High	146	32.52

^a^student, daily laborer, private work

### Obstetrics characteristics

Nearly three-fifths (57.94%) were multipara, and 46.10% had four and above ANC follow-ups. A considerable proportion of the participants, 9.35% and 13.14% experienced complications during pregnancy and delivery, respectively. Most (91.31%) were delivered through spontaneous vaginal delivery (SVD), two-fifth (38.53%) were born at home, and the pregnancy was planned for 78.84%. The majority (81.29%) did not receive postnatal care (PNC), exclusively breastfed for six months (63.7%), and began complementary feeding at six months (88.64%). over one-fifth (21.6%) had hazardous alcohol drinking habits, and 56.79% encountered DV. Furthermore, 83 (18.49%), 66 (14.7%), and 53 (11.8%) faced depression, anxiety, and stress symptoms, respectively (Table 2).

**Table 2:** Obstetrics characteristics of participants in Arba Minch health and demographic surveillance system sites, southern Ethiopia, 2022 (n = 449).

Variables	Category	Frequency	Percentage
Gravidity	1	80	17.82
	2–4	253	56.35
	≥ 5	116	25.84
Parity	Primiparity	81	18.12
	Multiparity	259	57.94
	Grand multiparity	107	23.94
^a^ANC follow-up	No	85	18.93
	1–3	157	34.97
	≥ 4	207	46.10
Pregnancy complication	Yes	42	9.35
	No	407	90.65
Labor and delivery complications	Yes	59	13.14
	No	390	86.86
Place of delivery	Home	173	38.53
	Health center	173	38.53
	Hospital	68	15.14
	Health post	35	7.80
Mode of delivery	^b^SVD	410	91.31
	Instrumental	26	5.79
	CS	13	2.90
Status of pregnancy	Planned	354	78.84
	Unplanned	95	21.16
^c^PNC follow-up	No	365	81.29
	1–3	53	11.80
	> 3	31	6.90
Counselled about breastfeeding techniques	Yes	194	43.21
	No	255	56.79
Exclusive breastfeeding for up to 6 months	Yes	286	63.70
	No	163	36.30
Start complementary feeding at six months	Yes	398	88.64
	No	51	11.36
Apply soothing techniques	Yes	378	84.19
	No	71	15.81
Fixed sleeping habit	Yes	179	39.87
	No	270	60.13
Hazardous alcohol drinking habit	Yes	97	21.60
	No	352	78.40
Domestic violence	Yes	255	56.79
	No	194	43.21
Common mental problems (^d^DAS)	Free of DAS	340	75.72
	Depression	27	6.01
	Anxiety	13	2.90
	Stress	10	2.23
	Depression & Anxiety	16	3.56
	Anxiety & stress	3	0.67
	Depression & Stress	6	1.34
	Comorbid DAS	34	7.57

^a^antenatal care;

^b^spontaneous vaginal delivery;

^c^postnatal care;

^d^depression, anxiety, and stress

### Infant characteristics

The mean age of the infants was 9.16 (SD: 2.25) months. More than half (55.01%) were male, and 7.57% were born with low birth weight. Of the infants, 14 (3.12%), 22 (4.90%), 33 (7.35%), and 160 (35.63%) had congenital anomalies, faced fetal distress during delivery, experienced complications during the postpartum period, and visited health facilities for medical illness, respectively (Table 3).

**Table 3:** Infants’ characteristics in Arba Minch health and demographic surveillance system sites, southern Ethiopia, 2022 (n = 449).

Variables	Category	Frequency	Percentage
Sex	Male	247	55.01
	Female	202	44.99
Age in months	6–8	195	43.43
	9–10	88	19.60
	11–12	166	36.97
Birth weight	Low birth weight	34	7.57
	Normal	396	88.2
	Macrosomia	19	4.23
Congenital anomalies	Yes	14	3.12
	No	435	96.88
Fetal distress during delivery	Yes	22	4.90
	No	427	95.10
Encounter postpartum complications*	Yes	33	7.35
	No	416	92.65
Visit health facilities for medical illness	Yes	160	35.63
	No	289	64.37

*Sepsis, jaundice, birth asphyxia, skin problems, gastrointestinal problems

### Prevalence of infant regulatory problems

**Fig 1:**
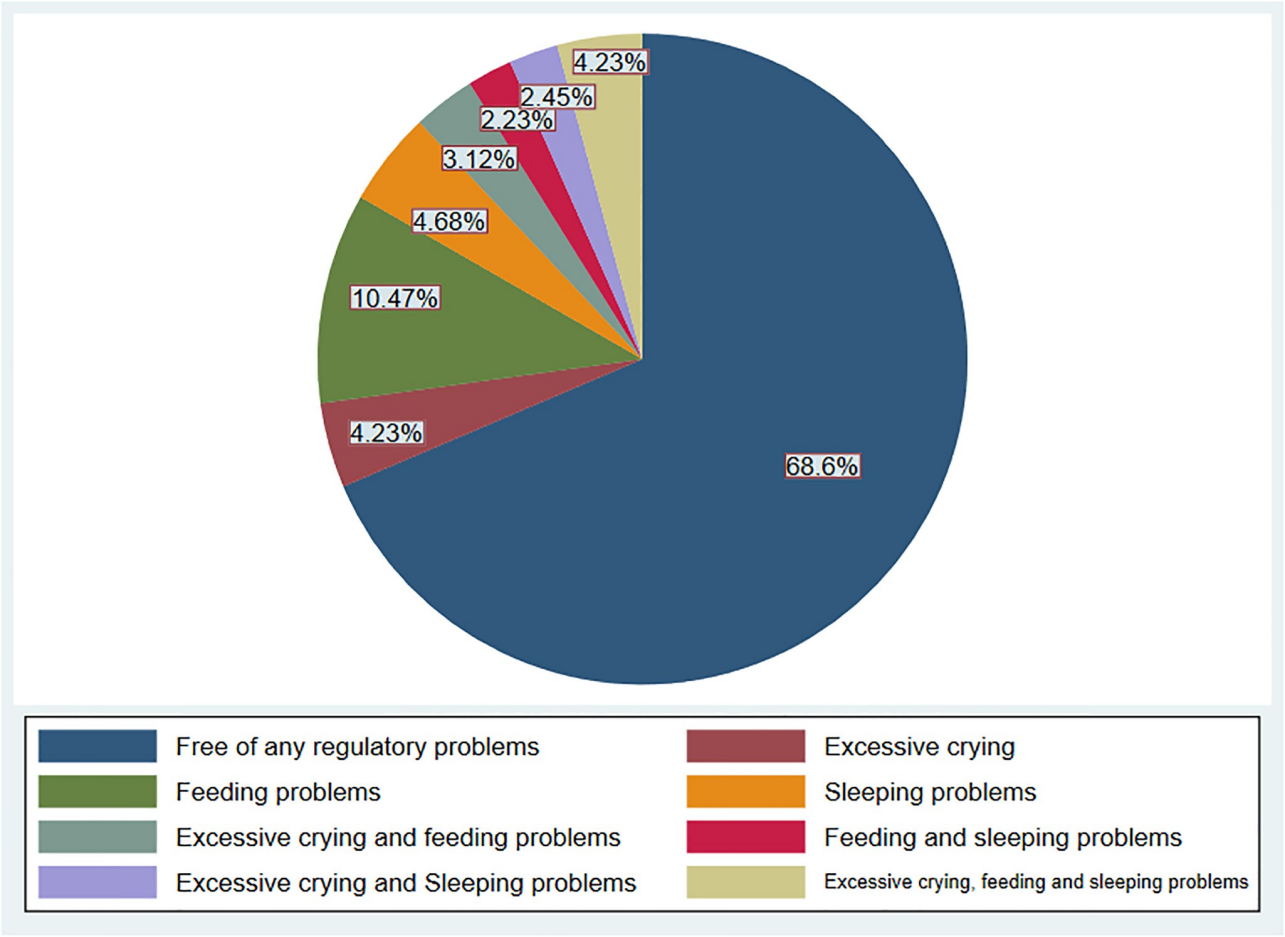
Prevalence of infant regulatory problems in Arba Minch health and demographic surveillance system sites, southern Ethiopia, 2022 (n = 449).

Of the infants, 63 [14.03%, 95% CI: 10.95, 17.59], 90 [20.04%, 95% CI: 16.44, 24.05], and 61 [13.59%, 95% CI: 10.55, 17.11] experienced excessive crying, feeding problems, and sleeping problems, respectively. Furthermore, 19 (4.23%) infants encountered comorbid infant RPs (Fig 1).

### Factors associated with excessive crying

After adjustment, educational status, PSS, feeding problems, and maternal DAS symptoms increase the likelihood of excessive crying. The odds of excessive crying were 2.5 times (AOR: 2.54, 95% CI: 1.22, 5.32) higher among infants whose mothers had primary education than infants whose mothers did not attend formal education. Maternal medium and high PSS decrease the odds of infant crying by 66.0% (AOR: 0.34, 95% CI: 0.13, 0.91) and 68.0% (AOR: 0.32, 95% CI: 0.12, 0.89), respectively compared to low PSS counterparts. Infant feeding problems increase the odds of excessive crying by five-fold (AOR: 5.0, 95%CI: 2.65, 9.45). The odds of excessive crying problems were 2.67 times (AOR: 2.67, 95% CI: 1.19, 5.98) higher among infants whose mothers experienced DAS symptoms than infants whose mothers were free of DAS symptoms (Table 4).

**Table 4:** Bivariable and multivariable analysis of factors associated with infant excessive crying in Arba Minch health and demographic surveillance system sites, southern Ethiopia, 2022 (n = 449).

Variables	Excessive crying		COR [95% CI]	AOR [95% CI]
	Yes, n (%)	No, n (%)		
**Educational status**				
No formal education	29 (10.28)	253 (89.72)	1	1
Primary education	24 (24.24)	75 (75.76)	2.79 (1.53, 5.08)	2.54 (1.22, 5.32) *
Secondary & above	10 (14.71)	58 (85.29)	1.50 (0.69, 3.26)	1.41 (0.55, 3.62)
**Family size**				
≤ 5	36 (15.93)	190 (84.07)	1.38 (0.80, 2.35)	1.22 (0.64, 2.36)
> 5	27 (12.11)	196 (87.89)	1	1
**Parity**				
Primiparity	20 (24.39)	62 (75.61)	2.43 (1.34, 4.41)	1.93 (0.89, 4.17)
Multiparity	43 (11.72)	324 (88.28)	1	1
**Infant age in months**				
6–8	21 (10.77)	174 (89.23)	1	
9–10	42 (16.54)	212 (83.46)	1.64 (0.94, 2.88)	1.36 (0.72, 2.57)
**Perceived social support**				
Poor	10 (32.26)	21 (67.74)	1	
Medium	31 (11.88)	230 (88.12)	0.28 (0.12, 0.66)	0.34 (0.13, 0.91) *
Good	22 (14.01)	135 (85.99)	0.34 (0.14, 0.82)	0.32 (0.12, 0.89) *
**Feeding problems**				
Yes	33 (36.67)	57 (63.33)	6.35 (3.59, 11.21)	5.0 (2.65, 9.45) *
No	30 (8.36)	329 (91.64)	1	
**Domestic violence**				
Yes	46 (18.04)	209 (81.96)	2.29 (1.27, 4.14)	1.98 (0.98, 3.96)
No	17 (8.76)	177 (91.24)	1	
**Maternal common mental problems** (DAS^a^)				
No	34 (10.0)	306 (90.0)	1	1
Single	14 (28.0)	36 (72.0)	3.5 (1.72, 7.13)	2.67 (1.19, 5.98) *
Multiple DAS	15 (25.42)	44 (74.58)	3.07 (1.55, 6.09)	1.85 (0.83, 4.16) *
**Alcohol abuse**				
Yes	23 (20.91)	87 (79.09)	1.98 (1.12, 3.48)	1.24 (0.63, 2.45)
No	40 (11.80)	299 (88.20)	1	1
**Exclusive breastfeeding for up to 6 months**				
Yes	36 (12.59)	250 (87.41)	1	
No	27 (16.56)	136 (83.44)	1.38 (0.80, 2.37)	1.29 (0.69, 2.43)

^a^depression, anxiety, and stress; Hosmer-Lemeshow: 0.4069

### Factors associated with feeding problems

In the multivariable analysis, family size, excessive crying, sleeping problems, maternal DAS symptoms, alcohol abuse, and late initiation of complementary feeding were associated with infant feeding problems. The odds of infant feeding problems were 1.82 times (AOR: 1.82, 95% CI: 1.03, 3.22) higher among infants with a family size of above five compared to infants with a family size of less than or equal to five. Infant excessive crying and sleeping problems increase the odds of infant feeding problems by fourfold (AOR: 3.76, 95% CI: 1.85, 7.65) and twofold (AOR: 2.29, 95% CI: 1.13, 4.65), respectively. The odds of infant feeding problems were 2.25 times (AOR: 2.25, 95% CI: 1.05, 4.82) and 3.42 times (AOR: 3.42, 95% CI: 1.64, 7.11) higher among infants whose mothers experienced single and comorbid DAS symptoms compared to infants whose mothers were free of any DAS symptoms. Alcohol abuse increases the odds of infant feeding problems by twofold (AOR: 1.89, 95% CI: 1.04, 3.42). Furthermore, the odds of infant feeding problems were three times (AOR: 2.67, 95% CI: 1.22, 5.88) higher among mothers who did not initiate complementary feeding at six months (Table 5).

**Table 5:** Bivariable and multivariable analysis of factors associated with infant feeding problems in Arba Minch health and demographic surveillance system sites, southern Ethiopia, 2022 (n = 449).

Variables	Feeding problems		COR [95% CI]	AOR [95% CI]
	Yes, n (%)	No, n (%)		
**Family size**				
≤ 5	39 (17.26)	187 (82.74)	1	1
> 5	51 (22.87)	172 (77.13)	1.42 (0.89, 2.26)	1.82 (1.03, 3.22) *
**Parity**				
Primiparity	20 (24.39)	62 (75.61)	1.37 (0.78, 2.41)	1.13 (0.54, 2.36)
Multiparity	70 (19.07)	297 (80.93)	1	1
**Pregnancy status**				
Planed	66 (18.64)	288 (81.36)	1	
Unplanned	24 (25.26)	71 (74.74)	1.48 (0.86, 2.52)	1.08 (0.56, 2.07)
**Experience pregnancy complications**				
Yes	13 (30.92)	29 (69.05)	1.92 (0.95, 3.87)	1.90 (0.82, 4.39)
No	77 (18.92)	330 (81.08)	1	
**Infant age in months**				
6–8	33 (16.92)	162 (83.08)	1	1
9–10	57 (22.44)	197 (77.56)	1.42 (0.88, 2.29)	1.61 (0.91, 2.85)
**Perceived social support**				
Poor	7 (22.58)	24 (77.42)	0.88 (0.35, 2.21)	0.52 (0.16, 1.65)
Medium	44 (16.86)	217 (83.14)	0.61 (0.38, 1.00)	0.58 (0.33, 0.1.33)
Good	39 (24.84)	118 (75.16)	1	
**Infant excessive crying**				
Yes	33 (52.38)	30 (47.62)	6.35 (3.59, 11.21)	3.76 (1.85, 7.65) *
No	57 (14.77)	329 (85.23)	1	
**Sleeping problems**				
Yes	29 (47.54)	32 (52.46)	4.86 (2.74, 8.61)	2.29 (1.13, 4.65) *
No	61 (15.72)	327 (84.28)	1	1
**Domestic violence**				
Yes	64 (25.10)	191 (74.90)	2.17 (1.31, 3.57)	1.27 (0.69, 2.33)
No	26 (13.40)	168 (86.60)	1	1
**Maternal common mental problems** (DAS^a^)				
No	50 (14.71)	290 (85.29)	1	1
Single	15 (30.0)	35 (70.0)	2.49 (1.27, 4.88)	2.25 (1.05, 4.82) *
Multiple DAS	25 (42.37)	34 (57.63)	4.26 (2.35, 7.75)	3.42 (1.64, 7.11) *
**Alcohol abuse**				
Yes	34 (30.91)	76 (69.09)	2.26 (1.38, 3.71)	1.89 (1.04, 3.42) *
No	56 (16.52)	283 (83.48)	1	
**Exclusive breastfeeding for up to 6 months**				
Yes	48 (16.78)	238 (83.22)	1	1
No	42 (25.77)	121 (74.23)	1.72 (1.08, 2.75)	1.25 (0.71, 2.20)
**Initiate complementary feeding at six months**				
Yes	75 (18.84)	323 (81.16)	1	1
No	15 (29.41)	36 (70.59)	1.79 (0.93, 3.45)	2.67 (1.22, 5.88) *
**Postnatal care utilization**				
Yes	11 (13.10)	73 (86.90)	1	1
No	79 (21.64)	286 (78.36)	1.83 (0.93, 3.62)	1.20 (0.53, 2.73)

^a^depression, anxiety, and stress; Hosmer-Lemeshow: 0.3288

### Factors associated with sleeping problems

After controlling for confounding variables; educational status, feeding problems, and maternal DAS symptoms independently increase the risk of infant sleeping problems. The odds of infant sleeping problems were 2.35 times (AOR: 2.35, 95% CI: 1.16, 4.77) higher among infants whose mothers had attended primary education than infants whose mothers did not attend formal education. Infant sleeping problems were 3.47 times (AOR: 3.47, 95%CI: 1.86, 6.48) higher among infants with feeding problems than their counterparts. Furthermore, the odds of infant sleeping problems were 3.23 times (AOR: 3.23, 95% CI: 1.53, 6.84) higher among infants whose mothers experienced comorbid DAS symptoms (Table 6).

**Table 6:** Bivariable and multivariable analysis of factors associated with infant sleeping problems in Arba Minch health and demographic surveillance system sites, southern Ethiopia, 2022 (n = 449).

Variables	Sleeping problems		COR [95% CI]	AOR [95% CI]
	Yes, n (%)	No, n (%)		
**Educational status**				
No formal education	32 (11.35)	250 (88.65)	1	
Primary education	21 (21.21)	78 (78.79)	2.10 (1.15, 3.86)	2.35 (1.16, 4.77) *
Secondary & above	8 (11.76)	60 (88.24)	1.04 (0.46, 2.38)	1.0 (0.38, 2.61)
**Parity**				
Primiparity	17 (20.73)	65 (79.27)	1.92 (1.03, 3.57)	1.74 (0.83, 3.67)
Multiparity	44 (11.99)	323 (88.01)	1	
**Feeding problem**				
Yes	29 (32.22)	61 (67.78)	4.86 (2.74, 8.61)	3.47 (1.86, 6.48) *
No	32 (8.91)	327 (91.09)	1	1
**Maternal common mental problems** (DAS^a^)				
No	35 (10.29)	305 (89.71)		
Single	8 (16.0)	42 (84.0)	1.66 (0.72, 3.82)	1.23 (0.50, 3.03)
Multiple DAS	18 (30.51)	41 (69.49)	3.83 (1.99, 7.37)	3.23 (1.53, 6.84) *
**Alcohol abuse**				
Yes	21 (19.09)	89 (80.91)	1.76 (0.99, 3.15)	1.31 (0.67, 2.55)
No	40 (11.80)	299 (88.20)	1	
**Domestic violence**				
Yes	39 (15.29)	216 (84.71)	1.41 (0.81, 2.47)	0.90 (0.54, 1.74)
No	22 (11.34)	172 (88.66)	1	1
**Exclusive breastfeeding for up to 6 months**				
Yes	31 (10.84)	255 (89.16)	1	
No	30 (18.40)	133 (81.60)	1.86 (1.08, 3.20)	1.77 (0.96, 3.26)
**Initiate complementary feeding at six months**				
Yes	51 (12.81)	347 (87.19)	1	1
No	10 (19.61)	41 (80.39)	1.66 (0.78, 3.52)	1.54 (0.66, 3.56)
**Postnatal care utilization**				
Yes	8 (9.52)	76 (90.48)	1	1
No	53 (14.52)	312 (85.48)	1.61 (0.74, 3.54)	1.32 (0.54, 3.21)

^a^depression, anxiety, and stress; Hosmer-Lemeshow: 0.1694

## Discussion

This study found that 14.03% of infants experienced excessive crying, 20.04% had feeding problems, and 13.59% struggled with sleeping difficulties. Maternal primary education, medium and high PSS, feeding problems, and maternal DAS symptoms were significantly associated with excessive crying. Likewise, a family size of above five, excessive crying, sleeping problems, single and comorbid DAS symptoms, alcohol abuse, and late initiation of complementary feeding increase the odds of infant feeding problems. Furthermore, maternal primary education, excessive crying, and maternal comorbid DAS symptoms were significantly associated with infant sleeping problems.

The current study demonstrated that 14.03% of infants experience excessive crying. This finding is consistent with the studies in Hungary (15.0%) [37] and Denmark (14.3%) [3], and lower than the studies in Australia (27.4%) [5] and Denmark (18.1%) [36]. This study also found that 20.04% of infants experience feeding problems. This finding is in line with the study in Denmark, 19.8% [3], higher than the study in Hungary (16.0%) [37], and lower than the studies conducted in Australia (25.2%) [5] and Germany (36.4%) [38]. Furthermore, this study revealed that 13.59% of infants encounter sleeping problems. This finding is comparable to the studies in the US (13%) [44] and Germany (12.2%) [38], higher than the studies in Hungary (10.0%) [37], Denmark (9.6%) [3], and the US (10%) [45]. On the other hand, this finding is lower than the studies in Australia (38.5%) [5] and Turkey (35.8%) [46].

Variations in operational definitions and measurement scales used to assess RPs, such as the Baby-DIPS in this study, could account for the discrepancies [35]. In contrast, previous studies used yes-or-no questions to ask mothers about their infant’s crying experiences, feeding and sleeping problems during the previous weeks, and different time points in the postpartum period. Participants’ socioeconomic status, level of education, and cultural background contribute to these variations. Furthermore, previous studies have been restricted to developed countries with advanced health-care systems that offer parenting advice to improve parents’ perceptions of infant RPs [3, 19, 20].

In this study, infants whose mothers attended primary school had a higher risk of excessive crying than infants whose mothers had no formal education. This finding contradicts with the study demonstrating that low maternal education increases the likelihood of infant RPs [3]. One justification is that high maternal schooling improves the perception of the mother to detect RPs. Another explanation is that women with a prominent level of education are more likely to be employed or engaged in economic activities, which results in shortage of time to properly care their infants. This might result in psychosocial stress and mental health issues, which can result in excessive crying in infants.

Our study found that maternal medium and high PSS decrease the odds of excessive crying. This is probably because good social support helps the mother treat her child effectively. Alexander and coauthors reported that high maternal social support during pregnancy and postpartum decreases the risk of infant fussiness/colic [47]. Ickes and colleagues also revealed that high social support improves child feeding practice [48]. In contrast, women who perceive themselves as tense and have low self-esteem increase the odds of infant RPs [5].

This study demonstrated that feeding problems increase the likelihood of excessive infant crying by fivefold. There is also an inverse relationship between excessive crying and feeding problems, which indicates RPs occurred concurrently, and early RPs are associated factors for late RPs [3]. This finding indicates that infant RPs can occur as isolated or comorbid, with one being a risk factor for the other [9]. Due to the study design, we were unable to assess the cause-effect relationship. As a result, scholars must fill this vacuum with better methodological approaches such as prospective study designs.

This study showed that a family size above five increases the risk of infant feeding problems by twofold. This is probably because as the number of children increases, the resources and time invested for each child decreases, which has a negative impact on child outcome [49]. Furthermore, low parenting self-efficacy is connected with poor execution of infant feeding practices, which can lead to RPs [50]. Thus, parents should be advised to have consistent feeding methods to promote healthy eating habits.

In this study, excessive crying and sleeping problems increased the odds of infant feeding problems by fourfold. These findings are consistent with the studies that revealed excessive crying hampers breastfeeding [14], decreases mother-infant attachment, and increases stress that affects the infant’s feeding habits [18]. This is likely due to the fact that isolated or comorbid RPs are common among infants with prematurity, fetal abnormalities, and fecal microbiota [7, 29].

This study revealed that alcohol abuse doubles the odds of feeding problems. The possible rationale is that alcohol increases the risk of maternal mental health problems, which further impairs the mother’s parenting skills, including infant feeding practice [51]. Furthermore, alcohol passes through the breast milk and affects the infant’s healthy development, which may lead to RPs [52].

Our study found that infants whose mothers attended primary school had a higher risk of sleeping problems than infants whose mothers did not attend formal education. This finding contradicts with a study that revealed low maternal education increases the risk of combined RPs in late infancy [3]. One explanation is that mothers with a prominent level of education have a better perception to detect their infant’s problematic sleeping pattern [46].

According to this study, mothers who did not initiate complementary feeding at six months are three times higher risk to experience infant feeding problems. On the other hand, a study conducted in Germany demonstrated that breastfeeding decreases the odds of isolated feeding problems by 49% [7]. This study also demonstrated that infant feeding problems triple the likelihood of sleeping problems. This finding is consistent with the findings of Messayke et al. (2021), who discovered that early initiation of baby cereals, thickened formula, and breastfeeding over six months are associated with poor sleep [53]. Field (2017) also ascribed that feeding problems are factors associated with isolated infant sleeping problems [54]. Furthermore, Schmid and colleagues revealed that breastfeeding increases the odds of isolated sleeping problems by fivefold [7]. The possible justification is that timely, adequate, safe, and proper initiation of complementary feeding around six month and continuation of breastfeeding up to two years are mandatory not only to ensure infant nutritional need but also to minimize the risk of RPs. Moreover, infant RPs are inextricably linked and affect one another. Therefore, parents should be advised on infant and young child feeding practices.

Our study revealed that maternal DAS symptoms increase the likelihood of all infant RPs. This finding aligns with previous studies, which consistently found that maternal depression and anxiety are linked to infant RPs [20, 36, 38, 46, 55]. The possible justification is that maternal depression has several health impacts: sleeping, emotional, social, and behavioral development problems. Furthermore, it impairs mother-infant interaction, breastfeeding, and maternal role [56]. Similarly, anxiety has many negative consequences, including breastfeeding difficulties and low self-efficacy [57]. Furthermore, stress impacts infant nutrition, maternal-child bonding, and sleep. Interventions targeting maternal mental health issues throughout pregnancy and the postpartum period are therefore essential for enhancing infant care and mother-infant attachment.

In general, evidence on infant RPs in developing countries, including Ethiopia, is limited. As a result, the findings of this study can serve as a foundation for further studies and interventions aimed at reducing RPs and their immediate and long-term complications. This study reports the burden of excessive crying, feeding, and sleeping problems, which is imperative for clinicians to treat and initiate counselling services for parents. In addition, the study identifies the factors associated with RPs. Comorbid DAS symptoms increase all forms of infant RPs so health care providers should include psychological interventions and coping strategies for parents. Lactating women must also be advised to avoid excessive alcohol consumption due to its impact on feeding problems and other RPs. Emphasis should be given to timely initiation of complementary feeding and improving social support as they are imperative to decrease the risk of feeding problems and excessive crying, respectively. Furthermore, infant RPs are still one of the most overlooked problems in Ethiopia, thus this study will be used for the scientific community to launch interventions and conduct other studies with a better methodological approach.

This study had several strengths, including using a reliable data collection tool, electronic data collection using ODK tools, and data collected by senior AM-HDSS data collectors. Despite its strength, this study may encounter the following limitations: due to limited studies in developing nations, the discussion was merely written compared to developed countries. Social desirability bias may underestimate the overall prevalence of infant RPs because such problems are culturally linked with poor parenting skills. Recall bias may also be introduced for infant and prenatal-related variables such as frequency of ANC, PNC, and birth weight. In addition, the study did not include mothers with severe mental illnesses, which may underestimate the actual prevalence of RPs. Moreover, the study only included the rural and semi-urban areas, and generalizability to the metropolitan settings was not possible. Lastly, the cross-sectional study design did not allow us to investigate the cause-effect relationship between various variables. Scholars are encouraged to conduct a prospective study with better study designs in different settings.

## Conclusions

Nearly one-third of the infants experienced RPs; one out of every seven infants had excessive crying and sleeping problems, and one out of every five had feeding problems. Maternal DAS symptoms increase the likelihood of excessive crying, feeding, and sleeping problems. Attending primary education, high PSS, and feeding problems increase the odds of excessive crying. Likewise, large family sizes, excessive crying, sleeping problems, alcohol abuse, and late initiation of complementary feeding increase the odds of feeding problems. Additionally, primary school attendance and excessive crying increase the likelihood of sleeping problems. Hence, it is crucial to provide counseling to parents regarding coping skills and suitable calming measures for infant RPs. Furthermore, early detection and treatment of maternal DAS and continuous emotional support are crucial to minimize the incidence of infant RPs.

## Supporting information

S1 File Data collection tool. (DOCX)

S1 Checklist STROBE statement for a manuscript entitled: Regulatory problems and associated factors among infants in Arba Minch health and demographic surveillance system sites, southern Ethiopia. (DOCX)

## Abbreviations

DAS: Depression, Anxiety, and Stress
DASS-21: Depression, Anxiety, and Stress Scale 21
DIPs: Diagnostic Interview for Regulatory Problems
MSPSS: Multidimensional Scale of Perceived Social Support
ODK: Open Data Kit
PSS: Perceived Social Support
RPs: Regulatory Problems
