# Supplementary material for: Regulatory problems and associated factors among infants in Arba Minch health and demographic surveillance system sites, southern Ethiopia
Source: PLoS One. 2024 Jun 18;19(6):e0305722. doi: 10.1371/journal.pone.0305722 (PMC11185483; doi:10.1371/journal.pone.0305722)
Supplement: S1 File — (DOCX) [file pone.0305722.s001.docx]

**Data collection tool to assess infant regulatory problems and associated factors**

| Name of the kebele: ________________________________ | | | |
| --- | --- | --- | --- |
| Household number: ______________________________ | | | |
| **S/N** | **Question** | **Response** | **Skip** |
| **Part I: Socio-demographic Characteristics** | | | |
|  | How old are you? ( in completed years) | **______________** |  |
|  | What is your level of education? | 1. Unable to read and write 2. Able to read and write 3. Primary (1-8) 4. Attend grade 9-12 5. College and above |  |
|  | What is your husband’s level of education? | 1. Unable to read and write 2. Able to read and write 3. Attend Primary (1-8) 4. Attend grade 9-12 5. College and above |  |
|  | What is your marital status? | 1. Single 2. Married 3. Divorced 4. Widowed 5. Married but living apart |  |
|  | What is your occupation? | 1. House wife 2. Merchant 3. Government employee 4. Non-governmental employee 5. Others (specify)_______ |  |
|  | What is the main occupation of your husband? | 1. Farmer 2. unemployed 3. Daily Laborer 4. Trader/Merchant 5. Government employee 6. Non-governmental employee 7. Others (specify)_______ |  |
|  | What is your residence? | 1. Urban 2. Rural 3. Semi-urban |  |

| 1. **Questions to assess the current family wealth (economic condition):** Could you tell me if you have the following in your house? | | |
| --- | --- | --- |
| **Asset type** | **Response** | |
| **Domestic animals** |  |  |
| Ox | No (0) | Yes (1) |
| Cow | No (0) | Yes (1) |
| Calf | No (0) | Yes (1) |
| Sheep | No (0) | Yes (1) |
| Goat | No (0) | Yes (1) |
| Horse | No (0) | Yes (1) |
| Donkey | No (0) | Yes (1) |
| Mule | No (0) | Yes (1) |
| Cock and Hen | No (0) | Yes (1) |
| **Durable assets** |  |  |
| Television | No (0) | Yes (1) |
| Radio | No (0) | Yes (1) |
| Electricity | No (0) | Yes (1) |
| Refrigerator | No (0) | Yes (1) |
| Conventional telephone | No (0) | Yes (1) |
| Mobile phone | No (0) | Yes (1) |
| Car | No (0) | Yes (1) |
| Motorcycle | No (0) | Yes (1) |
| Cycle | No (0) | Yes (1) |
| Cart | No (0) | Yes (1) |
| Gold, money | No (0) | Yes (1) |
| Ownership of owned living house | No (0) | Yes (1) |
| Ownership of agricultural land | No (0) | Yes (1) |
| **Productive assets** |  |  |
| Plough plow | No (0) | Yes (1) |
| Axe | No (0) | Yes (1) |
| Hoe | No (0) | Yes (1) |
| Shovel | No (0) | Yes (1) |
| Sickle | No (0) | Yes (1) |
| Modern beehive | No (0) | Yes (1) |
| Traditional beehive | No (0) | Yes (1) |
| **Housing characteristics** |  |  |
| Indoor plumping/ pipe water | No (0) | Yes (1) |
| Type of flooring | Earth/dung (0) | Cement/raw wood (1) |
| **Other household materials** |  |  |
| Bed | No (0) | Yes (1) |
| Table | No (0) | Yes (1) |
| Chair | No (0) | Yes (1) |
| Stove | No (0) | Yes (1) |

| **Part II: Obstetrics Characteristics** | | | | | |
| --- | --- | --- | --- | --- | --- |
|  | How old were you during your first pregnancy in completed years? | | _____________ |  | |
|  | Total number of pregnancies? | | _______ if 1 | **206** | |
|  | Do you have history of stillbirth? | | 1. Yes 2. No |  | |
|  | Do you history of newborn death within the first 28 day’s | | 1. Yes 2. No |  | |
|  | Do you have history of abortion? | | 1. Yes 2. No |  | |
|  | Did you experience any serious health problems related to the recent pregnancy? | | 1. Yes 2. No |  | |
|  | Did you experience any serious health problems during labor and delivery? | | 1. Yes 2. No |  | |
|  | What was the mode of delivery? | | 1. Svd 2. Instrument delivery 3. C/s |  | |
|  | Gestational age during delivery (in weeks) | | _______________ |  | |
|  | Does the pregnancy wanted/supported? | | 1. Yes 2. No |  | |
|  | Did you see anyone for antenatal checkup during pregnancy? | | 1. Yes 2. No | **301** | |
|  | How many ANC visits did you attend for this pregnancy? | | _____________ |  | |
| **Part III: Infant characteristics** | | | | | |
|  | Age of the infant in months | ________ | | |  |
|  | Sex | 1. Male 2. Female | | |  |
|  | Does the newborn encounter any problem during delivery? | 1. Yes 2. No | | |  |
|  | Does the newborn deliver with any congenital anomalies? | 1. Yes 2. No | | |  |
|  | If yes for Q.603 which congenital anomalies? | ________________ | | |  |
|  | Does the newborn deliver at term? | 1. Yes 2. No | | |  |
|  | Does the newborn encounter any problem after delivery? | 1. Yes 2. No | | |  |
|  | If yes for Q.606 which problem she/he encountered? Multiple answer is possible? | 1. Sepsis 2. Jaundice 3. Birth asphyxia 4. Skin problems 5. GI problems 6. Other specify______ | | |  |
|  | Does the newborn visit to health facilities for medical illness? | 1. Yes 2. No | | |  |
|  | If yes for Q.608 by which problem he /she admitted? | ______________ | | |  |
|  | Does the infant exclusively breastfeed up to six months? | 1. Yes 2. No | | |  |
|  | Does the newborn start complementary feeding at 6 months? | 1. Yes 2. No | | |  |
| **Part IV. Infant regulatory problems assessment** | | | | | |
| 1. **Excessive crying** | | | | | |

|  | Did your child cry or fuss for more than 3 hours/day? (Crying for ≥3 h/day) | 1. Yes 2. No |
| --- | --- | --- |
|  | How many days per week did your child cry or fuss for more than 3 hours/day? (on ≥3 days/week) | **__________** |
|  | How many consecutive weeks did this period of excessive crying last? (for ≥3 weeks consecutively) | **____________** |
| 1. **Feeding problems** | | |
|  | Were any of these behaviours typical for your child’s feeding/eating behaviours? sucking problems, swallowing problems, digestion problems, colics/flatulence, unpleasant reactions to food (regurgitation, excessive drooling, nausea, vomiting), bad appetite, child falls asleep, child gets distracted, child resists (against breast, bottle, nourishments), fidgeting, crying/fussing, frequent refusal to eat, picky eating habits, irritable while eating, or other (please indicate)?” (Any feeding problem(s) from a list of 16 possible problems) | 1. Yes 2. No |
|  | Did your child eat too little food resulting in a remarkable weight loss or no significant weight gain?” (failure-to-thrive,) | 1. Yes 2. No |
|  | How much did you worry about your child not growing enough? (mother worries a lot or very much about infant growth) | 1. Not at all 2. somewhat 3. a lot 4. very much |
|  | Did these difficulties or weight problems last for at least 4 weeks?” (for ≥4 weeks consecutively) | 1. Yes 2. No |
|  | Did your child have any diseases explaining the reduced food intake?”(Exclusion: Cases that were better attributable to a concurrent medical condition) | 1. Yes 2. No |
| 1. **Sleeping problems** | | |
|  | Did it ever happen that your child lay in bed and could not initiate sleep for a period of more than 1 hour? (Difficulties in initiating  Sleep) | 1. Yes 2. No |
|  | Did it ever happen that your child could not maintain sleep during the night? (difficulties in maintaining sleep) | 1. Yes 2. No |
|  | How many nights per week was your child unable to initiate/maintain sleep?” (for ≥3 nights/week) | **_________** |
|  | Did these difficulties in initiating/maintaining sleep last for at least 3 months?” (for ≥3 months consecutively) | 1. Yes 2. No |
|  | How much have your infant’s sleeping difficulties impaired your daily life?” | 1. Not at all 2. somewhat 3. a lot 4. very much |

| **Part V: Interpersonal Relations**: No matter how well a couple gets along, there are times when they disagree. Couples get annoyed with the other person, or just have spats or fights | | | | | | | | | | | | | | | | | | |
| --- | --- | --- | --- | --- | --- | --- | --- | --- | --- | --- | --- | --- | --- | --- | --- | --- | --- | --- |
| because they’re in a bad mood or tired of for some other reason. They also use many different ways of trying to settle their differences. I’m going to ask what you and your partner might feel when you have an argument. | | | | | | | | | | | | | | | | | | |
|  | How do you and your partner work out arguments? | | | | | 1. no difficulty 2. some difficulty 3. great difficulty | | | | | | | | | |  | | |
|  | In general, how do you describe your relationship? | | | | | 1. no tension 2. some tension 3. a lot of tension | | | | | | | | | |  | | |
|  | How is your partner treating you and the kids? | | | | | 1. always well 2. well most of the time 3. neutral 4. not well most of the time 5. never well | | | | | | | | | |  | | |
|  | Do you feel safe in your current relationship? | | | | | 1. always safe 2. safe most of the time 3. neutral 4. not safe most of the time 5. never safe | | | | | | | | | |  | | |
|  | Considering your current partners, friends, or any past partners or friends, are there anyone who is making you feel unsafe now? | | | | | 1. always safe 2. safe most of the time 3. neutral 4. not safe most of the time 5. never safe | | | | | | | | | |  | | |
| **Part VI: Multidimensional Scale of Perceived Social Support:** Indicate how you feel about each statement. 1) Very Strongly Disagree, 2) Strongly Disagree, 3) Mildly Disagree, 4) Neutral, 5) Mildly Agree, 6) Strongly Agree, and 7) Very Strongly Agree | | | | | | | | | | | | | | | | | | |
| **S/n.** | | | Items | | 1 | | 2 | 3 | 4 | | 5 | | | 6 | | | 7 | |
|  | | | There is a special person who is around when I am in need | |  | |  |  |  | |  | | |  | | |  | |
|  | | | There is a special person with whom I can share joys and sorrows | |  | |  |  |  | |  | | |  | | |  | |
|  | | | My family really tries to help me. | |  | |  |  |  | |  | | |  | | |  | |
|  | | | I get the emotional help & support I need from my family. | |  | |  |  |  | |  | | |  | | |  | |
|  | | | I have a special person who is a real source of comfort to me. | |  | |  |  |  | |  | | |  | | |  | |
|  | | | My friends really try to help me. | |  | |  |  |  | |  | | |  | | |  | |
|  | | | I can count on my friends when things go wrong. | |  | |  |  |  | |  | | |  | | |  | |
|  | | | I can talk about my problems with my family. | |  | |  |  |  | |  | | |  | | |  | |
|  | | | I have friends with whom I can share my joys and sorrows. | |  | |  |  |  | |  | | |  | | |  | |
|  | | | There is a special person in my life who cares about my feelings. | |  | |  |  |  | |  | | |  | | |  | |
|  | | | My family is willing to help me make decisions. | |  | |  |  |  | |  | | |  | | |  | |
|  | | | I can talk about my problems with my friends. | |  | |  |  |  | |  | | |  | | |  | |
| **Part VII: Depression Anxiety Stress Scale 21 (DASS_21_):** Please read each statement and circle a number 0, 1, 2 or 3 which indicates how much the statement applied to you **over the past week**. There are no right or wrong answers. Do not spend too much time on any statement.  The rating scale is as follows: 0. Did not apply to me at all 1. Applied to me to some degree, or some of the time 2. Applied to me to a considerable degree or a good part of time 3. Applied to me very much or most of the time  Put **x** in each cells of the respective category | | | | | | | | | | | | | | | | | | |
| Items | | | | | | | | | | 0 | | 1 | | | 2 | | | 3 |
|  | | I found it hard to wind down (s) | | | | | | | |  | |  | | |  | | |  |
|  | | I was aware of dryness of my mouth (a) | | | | | | | |  | |  | | |  | | |  |
|  | | I couldn’t seem to experience any positive feeling at all (d) | | | | | | | |  | |  | | |  | | |  |
|  | | I experienced breathing difficulty (e.g. excessively rapid breathing, breathlessness in the absence of physical exertion) (a) | | | | | | | |  | |  | | |  | | |  |
|  | | I found it difficult to work up the initiative to do things (d) | | | | | | | |  | |  | | |  | | |  |
|  | | I tended to over-react to situations (s) | | | | | | | |  | |  | | |  | | |  |
|  | | I experienced trembling (e.g. in the hands) (a) | | | | | | | |  | |  | | |  | | |  |
|  | | I felt that I was using a lot of nervous energy (s) | | | | | | | |  | |  | | |  | | |  |
|  | | I was worried about situations in which I might panic and make a fool of myself (a) | | | | | | | |  | |  | | |  | | |  |
|  | | I felt that I had nothing to look forward to (d) | | | | | | | |  | |  | | |  | | |  |
|  | | I found myself getting agitated (s) | | | | | | | |  | |  | | |  | | |  |
|  | | I found it difficult to relax (s) | | | | | | | |  | |  | | |  | | |  |
|  | | I felt down-hearted and blue (d) | | | | | | | |  | |  | | |  | | |  |
|  | | I was intolerant of anything that kept me from getting on with what I was doing (s) | | | | | | | |  | |  | | |  | | |  |
|  | | I felt I was close to panic (a) | | | | | | | |  | |  | | |  | | |  |
|  | | I was unable to become enthusiastic about anything (d) | | | | | | | |  | |  | | |  | | |  |
|  | | I felt I wasn’t worth much as a person (d) | | | | | | | |  | |  | | |  | | |  |
|  | | I felt that I was rather touchy (s) | | | | | | | |  | |  | | |  | | |  |
|  | | I was aware of the action of my heart in the absence of physical exertion (e.g. sense of heart rate increase, heart missing a beat) (a) | | | | | | | |  | |  | | |  | | |  |
|  | | I felt scared without any good reason (s) | | | | | | | |  | |  | | |  | | |  |
|  | | I felt that life was meaningless (d) | | | | | | | |  | |  | | |  | | |  |
| **Part VIII: History of Substance Abuse:** The fast alcohol screening test (FAST) scale | | | | | | | | | | | | | | | | | | |
|  | How often do you have six or more drinks on one occasion? | | | 1. Never 2. once or less per month 3. Monthly 4. weekly 5. Daily | | | | | | | | |  | | | | | |
|  | How often during the last year have you been unable to remember what happened the night before because you have been drinking? | | | 1. Never 2. once or less per month 3. Monthly 4. Weekly 5. Daily | | | | | | | | |  | | | | | |
|  | How often during the last year have you failed to do what was normally expected of you because of drinking? | | | 1. Never 2. once or less per month 3. Monthly 4. Weekly 5. Daily | | | | | | | | |  | | | | | |
|  | In the last year has a relative or friend or a doctor or other health worker been concerned about your drinking or suggested you cut down? | | | 1. Never 2. once or less per month 3. Monthly 4. Weekly 5. Daily | | | | | | | | |  | | | | | |
|  | Did you have history of smoking in the last 12 months? | | | 1. Yes 2. No | | | | | | | | |  | | | | | |
|  | Did you chew khat at least once in the last 12 months? | | | 1. Yes 2. No | | | | | | | | |  | | | | | |

**Thank You!!!**
