# Supplementary material for: Regulatory problems and associated factors among infants in Arba Minch health and demographic surveillance system sites, southern Ethiopia
Source: PLoS One. 2024 Jun 18;19(6):e0305722. doi: 10.1371/journal.pone.0305722 (PMC11185483; doi:10.1371/journal.pone.0305722)
Supplement: S1 Checklist — (DOCX) [file pone.0305722.s002.docx]

STROBE Statement for a manuscript entitled: Regulatory problems and associated factors among infants in Arba Minch health and demographic surveillance system sites, southern Ethiopia

|  | Item No. | Recommendation | Page  No. | Relevant text from manuscript |
| --- | --- | --- | --- | --- |
| **Title and abstract** | 1 | (*a*) Indicate the study’s design with a commonly used term in the title or the abstract | 1 | Cross-sectional study, mentioned in the abstract |
|  |  | (*b*) Provide in the abstract an informative and balanced summary of what was done and what was found | 1 & 2 | The prevalence of excessive infant crying, feeding, and sleeping problems was 14.03%, 20.04%, and 13.59%, respectively. |
| Introduction | | | |  |
| Background/rationale | 2 | Explain the scientific background and rationale for the investigation being reported | 3 & 4 | Although infant regulatory problems are the most common reason for a healthcare facility visit and a source of parental distress, there is a paucity of studies in developing countries, including Ethiopia. |
| Objectives | 3 | State specific objectives, including any prespecified hypotheses | 4 | To determine the prevalence of regulatory problems among infants  To identify factors associated with regulatory problems |
| Methods | | | |  |
| Study design | 4 | Present key elements of study design early in the paper | 5 | A community-based cross-sectional study |
| Setting | 5 | Describe the setting, locations, and relevant dates, including periods of recruitment, exposure, follow-up, and data collection | 5 & 7 | This study was conducted among infants in Arba Minch health and demographic surveillance sites from February 15 to March 15, 2022 |
| Participants | 6 | (*a*) *Cohort study*—Give the eligibility criteria, and the sources and methods of selection of participants. Describe methods of follow-up  *Case-control study*—Give the eligibility criteria, and the sources and methods of case ascertainment and control selection. Give the rationale for the choice of cases and controls  *Cross-sectional study*—Give the eligibility criteria, and the sources and methods of selection of participants | 5 & 6 | Those infants whose mothers/guardians had severe mental health problems and could not respond and mothers under six-month infants were excluded.  A simple random sampling technique was used to select the individual households with infants |
|  |  | (*b*) *Cohort study*—For matched studies, give matching criteria and number of exposed and unexposed  *Case-control study*—For matched studies, give matching criteria and the number of controls per case |  |  |
| Variables | 7 | Clearly define all outcomes, exposures, predictors, potential confounders, and effect modifiers. Give diagnostic criteria, if applicable | 6-8 | The tool comprises six parts: Example. Part four includes the outcome variable, infant regulatory problems (RPs) (excessive crying, sleeping problems, and feeding difficulty). |
| Data sources/ measurement | 8* | For each variable of interest, give sources of data and details of methods of assessment (measurement). Describe comparability of assessment methods if there is more than one group | 6-8 | Detail description of each variable with their measurement and scoring was included. Example. Infant RPs were assessed using diagnostic interviews for regulatory problems (Baby-DIPS). |
| Bias | 9 | Describe any efforts to address potential sources of bias | 8 | The training was given to the data collection team for two days.  Data were collected electronically using the open data kit  Regular supervision of the data collection process. |
| Study size | 10 | Explain how the study size was arrived at | 6 | The sample size was determined by Epi-Info 7 StatCalc using double population formula. |

Continued on next page

| Quantitative variables | 11 | Explain how quantitative variables were handled in the analyses. If applicable, describe which groupings were chosen and why | 8 | Simple frequencies, mean, and standard deviation were used |
| --- | --- | --- | --- | --- |
| Statistical methods | 12 | (*a*) Describe all statistical methods, including those used to control for confounding | 8 | Descriptive statistical analyses  Principal component analysis  Bivariable and multivariable regression |
|  |  | (*b*) Describe any methods used to examine subgroups and interactions | N/A |  |
|  |  | (*c*) Explain how missing data were addressed |  |  |
|  |  | (*d*) *Cohort study*—If applicable, explain how loss to follow-up was addressed  *Case-control study*—If applicable, explain how matching of cases and controls was addressed  *Cross-sectional study*—If applicable, describe analytical methods taking account of sampling strategy | N/A |  |
|  |  | (*e*) Describe any sensitivity analyses | N/A |  |
| Results | | | | |
| Participants | 13* | (a) Report numbers of individuals at each stage of study—eg numbers potentially eligible, examined for eligibility, confirmed eligible, included in the study, completing follow-up, and analysed | 9 | A total of 449 mother-infant pairs were involved, with a response rate of 99.5% |
|  |  | (b) Give reasons for non-participation at each stage | N/A |  |
|  |  | (c) Consider use of a flow diagram | N/A |  |
| Descriptive data | 14* | (a) Give characteristics of study participants (eg demographic, clinical, social) and information on exposures and potential confounders | 9-13 | Maternal socio-demographic characteristics  Obstetrics characteristics  Infant characteristics |
|  |  | (b) Indicate number of participants with missing data for each variable of interest | 10-12 | Mentioned in each table heading |
|  |  | (c) *Cohort study*—Summarise follow-up time (eg, average and total amount) | N/A |  |
| Outcome data | 15* | *Cohort study*—Report numbers of outcome events or summary measures over time | N/A |  |
|  |  | *Case-control study—*Report numbers in each exposure category, or summary measures of exposure | N/A |  |
|  |  | *Cross-sectional study—*Report numbers of outcome events or summary measures | 13 | Of the participants, 141 [31.40%] encountered at least one infant RPs, 63 [14.03%], 90 [20.04%], and 61 [13.59%] experienced excessive crying, feeding problems, and sleeping problems, respectively |
| Main results | 16 | (*a*) Give unadjusted estimates and, if applicable, confounder-adjusted estimates and their precision (eg, 95% confidence interval). Make clear which confounders were adjusted for and why they were included | 14-18 | The final model for each outcome variable addressed all the requirements mentioned here: Tables 5-7. |
|  |  | (*b*) Report category boundaries when continuous variables were categorized | N/A |  |
|  |  | (*c*) If relevant, consider translating estimates of relative risk into absolute risk for a meaningful time period | N/A |  |

Continued on next page

| Other analyses | 17 | Report other analyses done—eg analyses of subgroups and interactions, and sensitivity analyses | N/A |  |
| --- | --- | --- | --- | --- |
| Discussion | | | | |
| Key results | 18 | Summarise key results with reference to study objectives | 18 | In the current study, nearly one-third of the infants had RPs; a significant number of infants had excessive crying, feeding problems, and sleeping problems |
| Limitations | 19 | Discuss limitations of the study, taking into account sources of potential bias or imprecision. Discuss both direction and magnitude of any potential bias | 22 | Social desirability bias may underestimate the overall prevalence of infant regulatory problems.  Recall bias may also be introduced for infant and prenatal-related variables. |
| Interpretation | 20 | Give a cautious overall interpretation of results considering objectives, limitations, multiplicity of analyses, results from similar studies, and other relevant evidence | 18-22 | The findings of this study were compared with all the available similar study findings, and scientific justification was given for each. |
| Generalisability | 21 | Discuss the generalisability (external validity) of the study results | 21 | The findings of this study can serve as a foundation for further studies and interventions aimed at reducing RPs and their associated complications in the Arba Minch Zuria district in Ethiopia |
| Other information | |  | | |
| Funding | 22 | Give the source of funding and the role of the funders for the present study and, if applicable, for the original study on which the present article is based | 23 | Arba Minch University (www.amu.edu.et) funds this study. The funders had no role in study design, data collection, and analysis, decision to publish, or preparation of the manuscript. |

*Give information separately for cases and controls in case-control studies and, if applicable, for exposed and unexposed groups in cohort and cross-sectional studies.

**Note:** An Explanation and Elaboration article discusses each checklist item and gives methodological background and published examples of transparent reporting. The STROBE checklist is best used in conjunction with this article (freely available on the Web sites of PLoS Medicine at http://www.plosmedicine.org/, Annals of Internal Medicine at http://www.annals.org/, and Epidemiology at http://www.epidem.com/). Information on the STROBE Initiative is available at www.strobe-statement.org.
